# The impact of COVID-19 in children and adolescents with obesity in Brazil

**DOI:** 10.20945/2359-3997000000462

**Published:** 2022-04-19

**Authors:** Caroline Rosa Pelicciari, Thiago Olivetti Artioli, Carlos Alberto Longui, Osmar Monte, Cristiane Kochi

**Affiliations:** 1 Unidade de Endocrinologia Pediátrica São Paulo SP Brasil Unidade de Endocrinologia Pediátrica, Irmandade da Santa Casa de Misericórdia de São Paulo, São Paulo, SP, Brasil

**Keywords:** Obesity, weight gain, COVID-19

## Abstract

**Objective:**

The primary goal of the study was to evaluate weight gain in children and adolescents with obesity during the COVID-19 pandemic period, and compare it with the period before the pandemic.

**Subjects and methods:**

The sample comprised 68 children with obesity aged between 7 and 18 years, 30 (44.1%) boys and 38 (55.9%) girls, who were attended at the pediatric endocrinology clinic of the Irmandade da Santa Casa de Misericórdia de São Paulo, SP, Brazil. Weight gain in the sample in the pre-lockdown period (December 2, 2018 to March 11, 2020) was compared with that in the lockdown period (March 11, 2020 to February 21, 2021).

**Results:**

Approximately one year before the start of the pandemic period, the mean (SD) chronological age was 10.1 years old (± 2.4), and an average weight gain of 4.4 kg (± 4.8) was observed during the pre-lockdown period described. One year after the start of the pandemic, mean (SD) chronological age was 11.8 years old (± 2.4), and an average weight gain of 8.5 kg (± 7.6) was observed in the lockdown period described. When we compared the weight gain in the two periods, it was higher in the pandemic period, both in girls and boys (p = 0.013 and 0.035, respectively).

**Conclusion:**

The results of the study show that the period of social isolation adopted to mitigate the COVID-19 pandemic was associated with increased weight gain in the studied population, probably due to a reduction in physical activities and an increase in energy consumption.

## INTRODUCTION

The World Health Organization (WHO) declared the novel coronavirus disease-2019 (COVID-19) outbreak a global pandemic on March 11th, 2020, as the number of confirmed cases outside China multiplied at an alarming rate ( 1 ). During the pandemic, many countries enacted social distancing measures to contain the spread of the severe acute respiratory syndrome coronavirus 2 (SARS-CoV-2). Although these actions were critical to reduce the transmission of COVID-19, they also had a series of negative effects, including on the health of children. School closure was one of the measures chosen by many governments, especially in Brazil, where children have missed more than 41 weeks of school ( 2 ), while the average time in the world was 14 to 15 weeks ( 3 ).

Childhood obesity is one of Brazil's greatest public health challenges, and many obesity-related risk factors are more prevalent during school breaks, as children are exposed to environments which favor weight gain because of easier access to high sugar and fat food sources, and a reduced consumption of vegetables ( 4 ). Although a modest increase in physical activity has been reported during school holidays, the social distancing measures enforced during the COVID-19 pandemic forced children and adolescents to stay at home, greatly reducing opportunities for physical activities and exercise.

The risk of weight gain in children as a consequence of school closures during COVID-19 is a great concern, especially after such a long period of time. Overweight and obesity can have both short-term consequences, such as dyslipidemia, orthopedic problems, and insulin resistance, and long-term consequences in adulthood, such as obesity, cardiovascular diseases and higher cancer risk ( 5 ).

The World Obesity Federation predicts that in 2025, we will have 206 million children and adolescents ( 5 –19 years) with obesity, and in 2030, 254 million, around the world. This same study predicts that in 2030, in Brazil, we will have 7.6 million children and adolescents aged 5-19 years with obesity. Our country would occupy the 5th place, with the largest number of obese children and adolescents in the world ( 6 ).

The World Health Organization (WHO) created a goal so that in 2025 there is no increase in the prevalence of obesity in children and adolescents around the world. The same study predicts that Brazil has a 2% chance of reaching the goal recommended by the WHO ( 6 ).

Therefore, the primary goal of this study was to evaluate whether there was an increase in weight gain during the pandemic period compared to weight gain before the lockdown period, in children with obesity.

## SUBJECTS AND METHODS

This study comprised 68 children with obesity (BMI standard deviation score above +2) (BMI SDS, WHO, 2007) ( 7 ) aged between 7 and 18 years of both genders who were being monitored in the outpatient pediatric endocrinology clinic in São Paulo, Brazil. It was a descriptive study, using a convenience sample, that obtained data recorded in a retrospective analysis that compared weight gain before the lockdown period (between December 2^th^ 2018 and March 11^th^ 2020) with that during this period (March 11^th^ 2020 to February 21^th^ 2021). The exclusion criteria were secondary causes of obesity, such as endocrine causes (hypothyroidism, Cushing disease, growth hormone deficiency, hypothalamic obesity, pseudohypoparathyroidism), genetic causes (monogenic obesity and syndromic obesity) and those patients who had not signed the consent form or have not accepted the terms of the research. All patients before the pandemic period were attended in the outpatient clinic and were weighed.

Our clinic is located in a tertiary public hospital, that attends patients with endocrinological issues, that are referred from a general pediatrician. The majority of our population has low-income and students undergo public's schools. The patients who were attended here undergo to multidisciplinary team. For that reason, we applied the binge eating scale (BES) questionnaire, which has been properly validated for use in Portuguese, to all patients to identify those with binge eating disorder (BED). Individuals with a score greater than 17 were considered to have a BED. Some studies suggest that psychological changes such as depression, anxiety, and eating disorders can lead to an increased risk of therapeutic failure ( 8 , 9 ). Therefore, the identification and treatment of these alterations are important in the therapeutic planning of patients with obesity. Due to the high rates of COVID-19 in Brazil, many children were unable to attend regular in person follow-up at our outpatient clinic. Patients who were unable to visit our outpatient clinic received a web-based survey to collect weight data related to the lockdown period. The survey was sent to approximately 100 obese children between January 15 and February 21, 2021, and we received 40 completed responses. Data was also obtained from 28 patients who visited the outpatient clinic during this period. The weight data was expressed in kilograms and body mass index was expressed as standard deviation from the WHO growth curves for school-aged children and adolescents (BMI SDS, WHO, 2007) ( 7 ).

Statistical analysis was performed using SigmaStat 3.5 for Windows (Systat Software, San Jose, California). The correlation between the two variables was established by Pearson's coefficient. The analysis of the same variable between two distinct and independent groups was performed by T-test (parametric distribution) or by the Mann-Whitney U test (nonparametric distribution). The analysis of the same individual in different periods of time was performed by paired T test. A 95% confidence interval (CI) was used, and p < 0.05 was considered statistically significant.

The study was approved by the Ethics Committee of *Irmandade da Santa Casa de Misericórdia de São Paulo* (protocol 2.525.308) (CAAE: 82948218.3.0000.5479). Written informed consent from the parents or guardians was obtained and assent was obtained from each subject following the principles of the Declaration of Helsinki.

## RESULTS

A total of 68 children with obesity were included, 30 (44.1%) boys had a median (IQR) BMI Z-score +3.0 (2.7-3.3) and 38 (55.9%) girls had a median (IQR) BMI Z-score +2.9 (2.6-3.1). Approximately one year before the start of the pandemic period, in December 2018, the observed o mean (SD) chronological age was 10.1 years old (± 2.4), and the mean (SD) weight gain was 4.4 kg (± 4.8), between the period to December 2018 to March 2020. Five patients experienced weight loss. No correlation was found between weight gain or BMI SDS before the lockdown period and chronological age or gender.

Approximately one year after the start of the pandemic period, around February 2021, the observed mean (SD) chronological age was 11.8 years old (± 2.4), and the mean (SD) weight gain was 8.5 kg (± 7.6). Six patients experienced weight loss. No correlation was found between weight gain and BMI SDS before school restriction season, as well as weight gain and chronological age.

When we compared weight gain in the two periods, it was higher in the period after the start of the pandemic, both in girls and boys (p = 0.013 and 0.035, respectively). However, boys had a higher weight gain than girls during this period (p = 0.049) ( Table 1 ).

**Table 1:** Weight gain in the pre-lockdown period (between December 2^th^ 2018 and March 11^th^ 2020) and the lockdown period (March 11^th^ 2020 to February 21^th^ 2021) by gender

		Pre-lockdown period (December 2, 2018 to March 11, 2020)		Lockdown period (March 11, 2020 to February 21, 2021)
Gender	BMI Z-score #	Weight gain (kg) (±SD)	Gender	Weight gain (kg) (±SD)
Female (n = 23)	2.9 (2.6-3.1)	3.4 (4.5)	Female (n = 38)	7.2 (7.5)
Male (n = 20)	3.0 (2.7-3.3)	5.65 (4.9) *	Male (n = 30)	10.1 (7.7) *

* T test, p < 0.05.

# Expressed as median (p25-75).

We would like to reassure that we had made statistical analysis of the data of self-reported weight gain in the pandemic period, 8.8 kg (4.8-12.6), compared to the data that was measured in a regular follow-up in our clinic, 6.9 kg (4.5-9.5), and there is not statistically significant difference (p = 0.310). Thus, it was not a bias in our study and our findings can be extended to the general population with obesity.

Among our sample, 16 children (9 female and 7 male) were classified as having BED. No correlation was found between weight gain and the presence of BED between the two periods, with both groups showing higher weight gain during the lockdown period ( Table 2 ).

**Table 2:** Weight gain according to the presence or absence of BED

			Period before pandemic period (December 2th 2018 to March 11th 2020)	Period during COVID-19 pandemic (March 11th 2020 to February 21th 2021)
BED	BMI Z-score #	BES Value &	Weight gain (kg)& #	Weight gain (kg)& #
Positive (n = 16)	3.1 (2.8-3.3)	25.2 (5.1)	7.1 (5.9)	12.5 (5.6)
Negative (n = 52)	2.8 (2.5-3.1)	11.1 (3.3)	2.8 (1.2-5.3)	7.8 (4.0-10.8)

BES: binge eating score; BED: binge eating disorder.

& Expressed as mean (±SD).

# EXPRESSED as median (p25-75).

* T test, p<0.05.

** Mann-Whitney test, p < 0.05.

## DISCUSSION

This study provides a snapshot of Brazilian children and adolescents with obesity who have been patients of our outpatient pediatric endocrinology clinic since at least December 2018 and uses data from their medical records, and their responses to a survey carried out between January 15 and February 21, 2021 to assess changes in weight in the pre-pandemic and pandemic periods. To the best of our knowledge, this is the first study to investigate the immediate impact of the COVID-19 pandemic on weight gain in the Brazilian pediatric population with obesity.

In the adult population, many studies have shown that the COVID-19 pandemic has had a significant negative impact on lifestyle behaviors and increased mental health problems, disproportionally affecting individuals with obesity ( 10 , 11 ). A Brazilian cohort study, called NutriNet, analyzed fourteen thousand adults and found an average 2 kg weight gain in 19.7% of the subjects and a weight loss in 15,2% ( 12 ). However, there have been few studies published in pediatric population. A Turkish study reported weight gain in children after a 3-month quarantine period ( 13 ), and a recent study of The Children's Hospital of Philadelphia found that overall obesity prevalence increased from 13.7% (June to December 2019) to 15.4% (June to December 2020) ( 14 ).

Our study demonstrated weight gain in children with obesity, and that the weight gain was greater in the period after the start of the pandemic period, especially in boys. When we analyzed the weight gain in the population with binge eating disorder, to our surprise, no correlation was found; we expected a greater weight gain in the subjects with BED, but this did not happen. We believe that the weight gain we detected is related to the social distancing measures put into place to contain the spread of SARS-CoV-2, which meant that children had to stay at home. Moreover, many schools introduced virtual or online classes, which forced students to use screens. In addition, social media and virtual games/video-games were an ideal platform for children and adolescents to keep connected with their friends and to provide entertainment at home, which resulted in an even greater increase in screen time and contributed to the sedentary life style that we suggest was at least partly responsible for the weight gain observed in this study.

Besides that, we emphasize that stocking up shelf stable food items was clearly a necessity and helped minimize trips outside of the home, but those items were normally ultra-processed and calorie-dense foods, and it could be also responsible for the weight gain observed in this study. In addition, children and adolescents suffered from the psychosocial impact of quarantine. Studies show that more than one-third of adolescents reported high levels of loneliness during the pandemic period ( 15 ). There are well established links between loneliness and mental health issues, such as depression and anxiety. These health problems can lead to an increase in food intake, induced by the feeling of pleasure that the food provides, worsening weight gain. Increased weight gain can worsen the individual's body self-image and emotional status, thereby creating a vicious circle.

Our main concern is the future consequences of obesity in childhood, both in the short term, causing dyslipidemia, orthopedic problems, insulin resistance, among other conditions; and in the long-term increasing adulthood obesity, cardiovascular diseases, raised cancer risk and a range of other health issues.

The main limitation of the present study is its dependence on data from a self-reported questionnaire, which may lead to the use of inaccurate of data. However, given the conditions resulting from the pandemic, it was the only safe way to do this. Other limitations include the lack of food questionnaire, screen time, physical activity time data, but all of our patients have sedentary habits and they reported a reduction in physical activities in this pandemic period.

In conclusion, the results of our study show that the social isolation used as a method to mitigate the COVID-19 pandemic has potentially worsened obesity in the studied population and led to significant weight gain, probably due to an increase in sedentary activities, adverse mental issues and the greater consumption of high calorie food.

It is important to stress that parents, physicians, hospital authorities, schools, government and non-governmental organizations and other bodies can play important roles in helping to mitigate the weight gain observed in children and adolescents during the pandemic. As a possible solution, the practice of indoor physical activity should be encouraged, such as skipping, dancing, or helping with household chores; as should a decrease in the consumption of ultra-processed foods, and the time spent using screens, as well as providing greater psychological support for these children and adolescents.

## References

[1] Cucinotta D, Vanelli M (2020). WHO Declares COVID-19 a Pandemic. Acta Biomed.

[2] Peixoto S [Internet] (2020). Brasil é o único país que está há 41 semanas sem aula, diz sindicato.

[3] OECD (2020). Education at a Glance 2020: OECD Indicators.

[4] Wang YC, Vine S, Hsiao A, Rundle A, Goldsmith J (2015). Weight-related behaviors when children are in school versus on summer breaks: does income matter?. J Sch Health.

[5] Sahoo K, Sahoo B, Choudhury AK, Sofi NY, Kumar R (2015). Childhood obesity: causes and consequences. J Family Med Prim Care.

[6] Global Atlas on Childhood Obesity [Internet] World Obesity Federation.

[7] Onis M, Onyango AW, Borghi E, Siyam A, Nishida C, Siekmann J (2007). Development of a WHO growth reference for school-aged children and adolescents. Bull World Health Organ.

[8] Sutaria S, Devakumar D, Yasuda SS, Das S, Saxena S (2019). Is obesity associated with depression in children? Systematic review and meta-analysis. Arch Dis Child.

[9] Fox CK, Gross AC, Rudser KD, Foy AM, Kelly AS (2016). Depression, Anxiety, and Severity of Obesity in Adolescents: Is Emotional Eating the Link?. Clin Pediatr (Phila).

[10] Flanagan EW, Beyl RA, Fearnbach SN, Altazan AD, Martin CK, Redman LM (2021). The Impact of COVID-19 Stay-At-Home Orders on Health Behaviors in Adults. Obesity.

[11] Di Renzo L, Gualtieri P, Pivari F, Soldati L, Attinà A, Cinelli G (2020). Eating habits and lifestyle changes during COVID-19 lockdown: an Italian survey. J Transl Med.

[12] Costa CS, Steele EM, Leite MA, Rauber F, Levy RB, Monteiro CA (2021). Mudanças no peso corporal na coorte NutriNet Brasil durante a pandemia de COVID-19. Rev Saúde Pública.

[13] Baysun Ş, Akar MN (2020). Weight gain in children during COVID-19 quarantine period. J Paediatr Child Health.

[14] Jenssen BP, Kelly MK, Powell M, Bouchelle Z, Mayne SL, Fiks AG (2021). COVID-19 and changes in child obesity. Pediatrics.

[15] Mental Health Foundation (2020). Loneliness during Coronavirus.

